# Journalistic “Lagniape”—The Reading Notice Nuisance

**Published:** 1891-09

**Authors:** 


### JOURNALISTIC "LAGNIAPE;" THE "READING NOTICE" NUISANCE.

If there be any who do not know the meaning and significance of "lagniape" we will tell them. It is a word much in vogue in New Orleans and elsewhere amongst the Creoles, and signifies a bonus, a premium, something given for "good measure," or for good will; something "thrown in" when a purchase is made. So general is the custom in New Orleans that if an urchin be not rewarded by a stick of candy, or an apple, along with his purchase of a nickel's worth of soap or starch, for instance, he feels defrauded of his rights. The custom is recognized and adhered to by all the hucksters and grocers, market folks and retail dealers generally.

Whether known by that name or not, does not matter much,—it would smell as bad by any other,—the practice has invaded the realms of the medical journal, and is spreading to an alarming degree. True, the journals do not sell soap or starch, but they sell advertising space; and although they do not deal in candy and apples, they are, almost without exception, addicted to "taffy," and deal it out in hunks, more or less, according to circumstances. We mean—to be more explicit—that it has become an established custom, in accepting an advertisement, to give a "notice" of it in the editorial page, to "call attention" to it; and this custom has grown and spread until the "reading notice" is not only expected, but is considered a matter of right. We plead guilty to the charge; we are given to giving "taffy" as lagniape, like all the brethren of the medical press, and we do it *because it is the custom.* It has reached that stage where a journal dare not refuse to insert "reading notices" for his advertisers,—he will be reminded that all others do; and one cannot well afford to be an exception to a general rule, especially if the loss of patronage be the consequence. Six years ago, when this JOURNAL began its career, an advertiser, in sending "copy," would, perhaps, politely suggest that a little editorial notice would be acceptable,—some modestly do so now; a little later four notices a year was stipulated as part of the advertising contract;—a little later we were told by several large advertisers that such notices "are worth more than the advertisement;" so—like a stone rolling down the hill, this custom has increased, until to-day the advertiser looks upon the "reading notices" as a part of his due; he pays you for a page, a half, or quarter page advertment a year, or six or three months, and expects—some demand—a reading notice with every issue of the JOURNAL, as "lagniape."

These "reading notices" consist for the most part of from two to three lines of commendation, to two and three pages of an elaborate article—written by some doctor, in which the merits of some proprietary medicine are brought to the reader's attention; consist of short (or long) letters to the proprietor, commendatory of his preparations; and as they are usually inserted in the journal to which they are sent, without alteration, that journal is made to appear as endorsing it, or it is taken, or mistaken for the editorial utterances of the journal.

Well, like the little peach of the emerald hue, which brought so much grief and griping to "Johnny Jones and his sister Sue,"—it "grew and grew"—until it has attained to-day the proportions of a full grown and robust *nuisance.*

Many advertisers—our best patrons—send the JOURNAL regularly, every month, articles taken from other journals, or written especially for the purpose, and very politely, it is true, ask that they be "inserted in the next issue of your esteemed journal;" and, for one, we always insert them.

It is not the advertiser's fault; who can blame them for taking all we will give—in tLe way of "lagniape?" It is our own fault.

Publishers are themselves to blame for it; and if it continues to advance with the same speed and progress it has gained in the past two years, very soon there will be room in most of the smaller journals for—nothing else.

Why, sirs, to go through with the average journal, and then to fall upon two to five pages of "puffs"—for such they are—of nearly every article represented in the advertising pages, or what is worse, to have one's reading interrupted every few pages by such reading notices interspersed, reminds one of a circus and the side-shows;—in the midst of the performance, or just after it is over, the voice of the side-show man swells on the breeze in melodious tones, and they vie the one with the other in sounding the praises of their several specialties, and in endeavoring to catch the attention of the passers-by.

Now, how is this to be remedied? All must see the injustice of it, not only to the publisher, but to the subscribers who pay for the journal. It is as unreasonable as to expect an accoucheur who, having received a fee for a "delivery," is expected to visit the patient four to a dozen times afterwards, as "lagniape."

The Association of American Medical Editors will meet in St. Louis in October—for a special conference, it is announced. We have not been advised as to the special object of the conference, but we suggest that there is no subject connected with the medical publisher's business, which, in our judgment, demands more serious consideration than this very thing. Where is it to end? No one publisher likes to refuse a request of the kind from a prompt paying advertiser. We are all "clever fellows," and really like to be obliging—like to help make the ads. pay, if we can—for the interest of the patron and publisher are mutual, to some extent; but it is not right to do so at the expense of our own interests, or to trespass on the rights of the paying subscriber. We lose subscribers by too much of this sort of business; and seriously, in our humble judgment, the time has arrived when a halt should be called.

---

The New England Medical Journal appropriately celebrates its tenth anniversary by a "souvenir number," extra large —unusually interesting, and embelished with portraits of the medical lights of the present and immediate past. This is a commendable enterprise which we are sure Dr. Wiles' large circle of friends and readers will appreciate; it is most creditable.

